# Expert Systems and Robotics

**DOI:** 10.6028/jres.093.019

**Published:** 1988-06-01

**Authors:** T. L. Isenhour, J. C. Marshall

**Affiliations:** Department of Chemistry, Kansas State University, Manhattan, KS 66506; Department of Chemistry, Saint Olaf College, Northfield, MN 55057

In this paper, we will discuss the interface between expert systems and laboratory robotics. We will use examples from our recent research to illustrate how we are building an effective interface and indicate where we think this research will lead.

What are expert systems? As an operational definition we will adopt the following:

Expert Systems are a sub-specialty in artificial intelligence (AI). The term is generally understood to mean a “knowledge-based” or “knowledge-driven” system designed to represent and apply factual knowledge in specific, very limited areas of expertise.

In the early sixties, AI researchers attempted to simulate the complicated process of thinking by finding general methods for solving broad classes of problems. This proved too difficult and such attempts failed. In the early seventies the problem was reformulated to include careful attention to data structures but the emphasis was still on general knowledge. Progress was still limited. In the late seventies the problem was further refined to focus almost completely on the knowledge representation. The goal was to make intelligent programs by providing them with high quality, domain-specific knowledge about some limited problem area. This strategy is much like that used by a human expert and gives rise to the term “expert system.”

What domains are appropriate for expert system work? First and foremost, for the present state of expert systems technology the problem domain must be of limited scope. A majority of the people within the application field must agree that real experts do exist. The problem must be knowledge, not data, intensive. A problem is knowledge intensive if there is substantial variability in people’s ability to solve it. The problem must not require information from visual input. Multiple answers from the same input data can be handled but with limited success. Perhaps the best test of all for a potential candidate for expert system work is the so-called “telephone test.” If you have a problem and you are confident that if you called some known expert in the field, he or she could solve the problem for you in 30 minutes or less over the phone, then the problem is likely to be amenable to an expert system solution.

How do expert systems compare with human experts? The popular press has tended to be wildly optimistic about the present state of expert systems development. While many useful expert systems are available, they apply to very limited problem domains. In such domains expert systems can quickly provide answers that are consistent and objective. Expert systems can capture human expertise and make it permanent, widely available and easily portable. However, current expert systems lack the creativity and adaptability expected of a human expert.

How do expert systems work? Regardless of the details of the implementation, an expert system is a program driven by an inference engine towards a specific goal. It is, in the limit, a remarkably simple process involving a cleverly ordered series of “if tests.” A potential difficulty is, when the problem gets large and consequently the number of rule structures in the data base increases, an expert system can become difficult to modify, hard to debug and slow to execute.

## Expert Systems for Data Management

Chemists, particularly analytical chemists, have historically been very concerned with the storage and retrieval of information. Spectral libraries are a common example. There is presently much interest in the potential of so-called “smart data bases” [1]. The fundamental idea is that a data base is represented as a collection of executable statements rather than facts.

**Figure 1:**
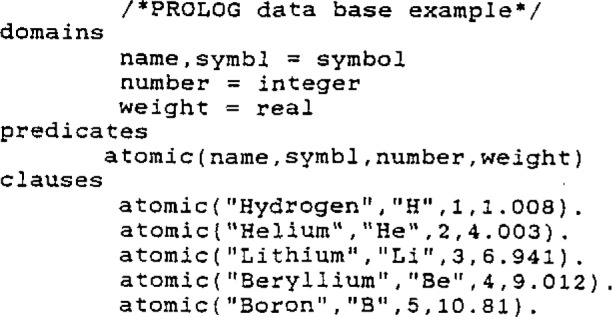
PROLOG data base example.

**Figure 2:**
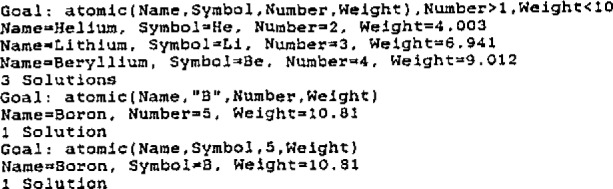
PROLOG data base interrogation examples.

The smart data base concept is a subtle strategy that can be illustrated with a trivial example involving the periodic table. The entries of the data base all conform to the PROLOG predicate ATOMIC and become executable statements; as such they are no longer passive facts. The PROLOG definitions and data base entries for a small part of this periodic table are shown in figure 1. In this example, apart from the definitions, there are no program statements other than the data base. A compelling advantage is that all of the features of the AI language used (in this case PROLOG) are available to form queries and the inference engine interrogates the file automatically. This is illustrated in figure 2.

## Methods Development Using Expert Systems and Robotics

A central theme in our research for the past several years has been the idea of the *Analytical Director*. Laboratory robots can carry out simple repetitive tasks, following an invariant set of rules. However, when a robot becomes a mechanical extension of a control program that has logic capability the whole becomes greater than the sum of the parts. The *Analytical Director* project is an expert system driven robot that combines knowledge about analytical chemistry with laboratory robotics. The system is presently capable, in a limited way, of designing procedures for analysis, testing and modifying such procedures, and finally archiving the modified procedure for future reference. The flexible library facilities of the *Analytical Director* are possible because of the “smart data” capabilities inherent in the logic based programming languages.

The current implementation of the *Analytical Director* is a Zymark robot running under control of the ARTS [2] software system, an expert system driven robotics language. The control computer is a simple PC.

**Figure 3:**
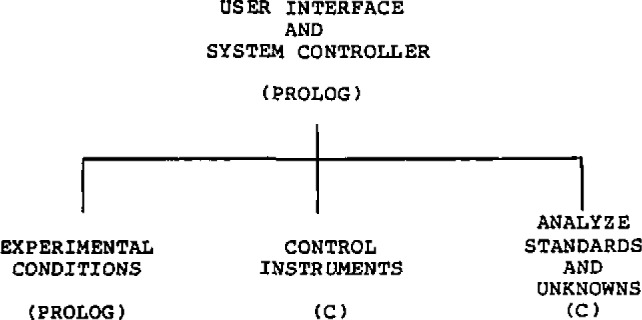
Complexometric titrations under expert system control.

To demonstrate an application of the *Analytical Director*, the development of a complexometric titration procedure [3] is shown as a flow chart in figure 3.

A successful complexometric titration requires that the conditions be adjusted so as to insure a conditional stability constant of about 1×10^5^. Choices to be made include the pH, the titrant, the masking agent or agents used and the method of endpoint detection. A vast literature exists on complexometric titrations. Some of this information is part of the knowledge base used by the ARTS system. The system not only starts with a knowledge base, but can continually update that knowledge base using results of experiments. The user is given the opportunity to specify some or all of the parameters that he/she wishes. The system will not override user input even though it may be wrong. The system will fill in missing user input from its knowledge base. The success or failure of a determination is stored by the system for future use.

Experimental results for the triplicate determination of Ni^+2^ by complexometric titration are shown in table 1 and compared with results obtained with manual titrations.

## Building Expert Systems from Chemical Data

One of the most difficult problems with expert system work is creating an efficient knowledge base. When the knowledge base gets large, it becomes imperative to create the most efficient possible production rules. The knowledge base used by an expert system can be most efficiently structured as a set of rules that describe the minimal decision tree spanning the data. The root node of this tree is the attribute of the data that minimizes the number of branches from the root. Each branch from the root node contains a different value of the root attribute and creates second level nodes. These second level nodes may be branched further using attributes different from the previous attributes used to split the data. The class attributes and values will occupy terminal nodes in the decision tree. If more than one attribute is used to describe the data, the decision tree will not be unique. As the number of attributes required to describe the data increases, the number of possible decision trees increases combinatorially.

For this task we have implemented the ID3 (iterative dichotomizer 3) algorithm [4–6], originally developed for organizing and optimizing chess end-game strategies.

The ID3 algorithm is based on information theory and uses the entropy of classification. The entropy of classification is a measure of the entropy resulting from classifying an object in a particular class. The algorithm will first determine the attribute to use for the root node of the tree so that the number of branches from the root node are minimum. Each branch from the root node represents a unique value of the root attribute. The algorithm is then applied recursively to all the second level nodes, and all subsequent nodes spawned by each of the second level nodes.

We have implemented the ID3 algorithm (7) in PROLOG so that it accepts classification data and determines an efficient set of rules spanning the data. The program will then produce a file of rules that can be used directly by an expert system as an efficiently ordered knowledge base.

A simple example of how this works uses the infrared data in table 2. These data are applied to identifying substituted benzenes from their infrared absorption spectra.

**Figure 4:**
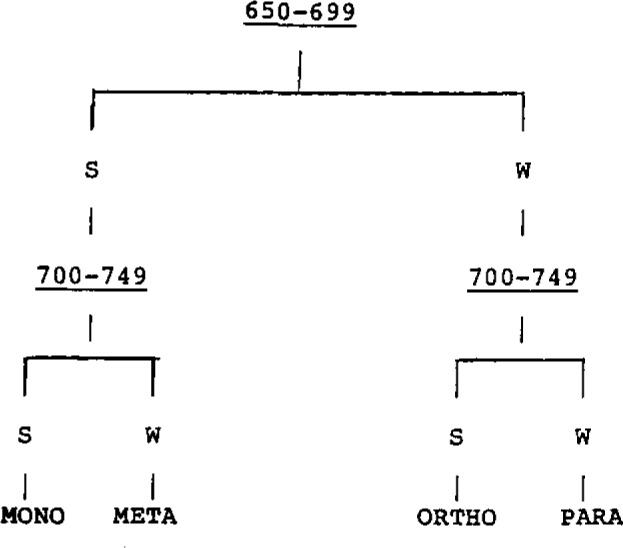
Decision tree generated from data in table 2.

There is enough information in the first two bands to answer the question. There is no information in the last two bands relevant to this question. From these data, the algorithm outputs the information tree shown in figure 4. Not shown is the set of syntactically correct PROLOG production rules generated by the program that span the tree in figure 4.

## Conclusion

The purpose of this research is the combination of logic programming with laboratory robotics. The goal of this research is the creation of the *Analytical Director*, an intelligent laboratory robotics system that will be able to develop, test and modify laboratory procedures without human supervision.

## Figures and Tables

**Table 1:** Comparison of expert system and human counterpart. Results for the titration of a Ni^2+^ solution using 0.1004M EDTA without an indicator. Absorbance data were collected at 480 nm

	Expert system	Human
Trial 1	0.1006	0.0981
Trial 2	0.1004	0.0981
Trial 3	0.1007	0.0983
Average	0.1005_7_	0.0981_7_
Standard deviation	0.0001_5_	0.0001_2_
%Standard deviation	0.15	0.12

**Table 2:** Infrared data for some substituted benzenes

Compound name	Degree of substitution	IR ranges in cm^−1^				
		650–699	700–749	750–799	800–849	950–899
toluene	MONO	S^a^	S	W	W	W
m-xylene	META	S	W	S	W	W
o-xylene	ORTHO	W	S	S	W	W
p-xylene	PARA	W	W	S	W	W

a S=strong; W = weak.
